# Topical insulin for refractory persistent corneal epithelial defects

**DOI:** 10.1038/s41598-024-63091-y

**Published:** 2024-05-30

**Authors:** Parisa Abdi, Reza Ghaffari, Nikoo Azad, Ahmed Alshaheeb, Golshan Latifi, Sahel Soltani Shahgoli, Hanieh Fakhredin

**Affiliations:** 1 Translational ophthalmology Research center, Farabi Eye Hospital, School of medicine, Tehran University of Medical Sciences, Tehran, Iran; 2 Ronald Reagan UCLA Medical Center, Los Angeles, CA USA; 3 Department of Plant Sciences, School of Biology, College of Science, University of Tehran, Tehran, Iran; 4 Ophthalmology Department, Farabi Eye Hospital, Tehran University of Medical Sciences, Tehran, Iran

**Keywords:** Corneal epithelium, Persistent epithelial defect, Corneal ulcer, Insulin, Diseases, Medical research

## Abstract

The aim was clinical evaluation of the efficacy of topical insulin eye drops in patients with refractory persistent epithelial defects (PEDs). This prospective non-randomized investigation was conducted to examine the efficacy of insulin eye drops in treating patients with PEDs that did not respond to conventional therapy. A total of twenty-three patients were included in the study, and they were administered insulin eye drops formulated as 1 U/mL, four times a day. The rate of epithelial defect resolution and time to complete corneal re-epithelialization were considered primary outcome measures. The relative prognostic impact of initial wound size and other parameters, including age, sex, smoking, diabetes, and hypertension were also analyzed. The results showed that during follow-up (maximum 50 days), a total of 16 patients (69.6%) achieved improvement. Insulin eye drops significantly reduced the corneal wounding area in 75% of patients with small epithelial defects (5.5 mm^2^ or less) during 20 days. Only 61% of patients with moderate epithelial defects (5.51–16 mm^2^) showed a significant recovery in 20–30 days. Also, 71% of patients with a defect size greater than 16 mm^2^, demonstrated a significant improvement in the rate of corneal epithelial wound healing in about 50 days. In conclusion topical insulin reduces the PED area and accelerates the ocular surface epithelium wound healing.

## Introduction

The cornea is the highly specialized transparent tissue of the anterior segment of the eye, which primarily provides the refractive interface for transmitting and focusing light entering the normal human eye. The surface of this avascular dome-shaped structure, composed mainly of water and collagen fibrils, is covered by layers of squamous non-keratinized epithelial cells^1^. The corneal epithelium, representing 10% of the corneal thickness, provides a smooth optical surface and forms a physical and immunological barrier composed of tight junctions, lateral gap junctions, and adherens junctions^2^. Under normal conditions, any  disruption in the integrity of the corneal epithelium usually triggers rapid wound healing responses in limbal epithelial stem cells (LESCs) through an intricate and dynamic interplay between several growth factors, cytokines, inflammatory mediators, and cellular signalings^3^. Failure or inability in rapid corneal re-epithelialization and restoration may contribute to persistent corneal epithelial defects (PEDs) that necessitate a completely different solution to avoid potentially sight-threatening consequences such as infections, melting stromal ulcers, and stromal opacity and neovascularization^4^.

Given the diverse etiological nature (neurotrophic cornea, surface trauma, defective epithelial adhesion, limbal stem cell deficiency, inflammation, and hereditary) and actual resistance to standard treatment options (lubrication, bandage contact lenses, punctual plugs, debridement and tarsorrhaphy), PEDs remain a serious treatment challenge for ophthalmologists^5^. In the last few years, novel non-invasive treatments including topical epidermal and insulin-like growth factors, topical fibronectin, autologous serum eye drops, and other hemoderivative products have demonstrated promising safety and efficacy in treating PEDs in several clinical trials^6–9^. Accordingly, topical insulin, a peptide hormone closely related to IGFs, has also shown to be a reliable and effective therapeutic modality for human ocular use in the setting of refractory corneal epithelial wound healing^10,11^. Despite the presence of insulin and its receptors in the human tear film and corneal epithelium, as well as the reported benefits such as convenient administration, high tolerability, and reduced complications in animal experiments, there is a limited number of prospective outcome studies that have examined the mechanisms by which topical insulin promotes the healing PEDs in humans^12–14^. This article aims to evaluate the clinical characteristics and outcomes of insulin eye drops as a treatment option for patients with PED that do not respond to conventional therapies.

## Materials and methods

In this prospective study, 23 PED patients were treated with off-label ophthalmic application of insulin eye drops at Farabi Eye Hospital from July 2019 to April 2021. The study was conducted with the approval of the medical Ethics committee of Tehran University of Medical Sciences, under the ethical code IR.TUMS.FARABIH.REC.1399.011, and adhered to the ethical guidelines outlined in the Declaration of Helsinki. The patients were prescribed insulin drops as a compassionate use treatment option for neurotrophic corneal ulcers that had not responded to standard treatment protocols.

Only patients who did not respond to intensive lubrication, bandage soft contact lenses, and other therapies targeting the underlying etiologies of PED within a 2-week period were included in the study and patients who had not completed at least two weeks of conventional treatments were excluded. To minimize further complications patients with concurrent infectious keratitis, trauma or acute chemical or thermal injury were also excluded. After explaining the purpose of this study and obtaining written informed consent from eligible patients, the following variables were recorded: age, gender, etiology of PED, duration since diagnosis, presence of hypertension, concurrent treatments, smoking status, best-corrected visual acuity (BCVA), and size and depth of the epithelial defect.

Prior to the application of topical insulin, the evaluation of the epithelial defect was conducted using fluorescein staining. Additionally, digital slit lamp photographs were captured on a daily basis throughout the duration of the healing process^15^. The epithelial defect size was estimated with ImageJ software using the digital images taken at regular intervals. All 23 patients received topical insulin eye drops (1 IU/mL) 4 times per day until recovery. The eye drops were prepared using soluble regular insulin (actrapid HM 1000 U, Novonordisk) in Balanced salt solution with a sterile technique^16^. Patients treated with topical insulin who showed any sign of deterioration or did not show a significant reduction in the wound’s size at the end of a 5-day treatment were excluded and placed in the non-responder category. These insulin-unresponsive patients underwent surgical or other invasive interventional procedures. The recorded data were analyzed using SPSS version 26.0 (Chicago: SPSS Inc.). We utilized Classification and Regression Trees (CRT) to determine optimal cutoff points for the size of the epithelial defect, enabling differentiation among the groups based on their final outcomes. Kaplan–Meier survival analysis and Cox regression analysis were performed to compare the time-to-heal and assess the relative prognostic impact of various factors such as initial wound size, age, gender, smoking habits, diabetes and hypertension. Descriptive statistics were employed to summarize the characteristics of the patients and their clinical outcomes. P values of less than 0.05 were considered statistically significant.

## Results

In this clinical investigation, a total of 23 eyes belonging to twenty-three patients (10 females and 13 males; average age of 56.6 years) with PED were examined. The patients received treatment in the form of insulin administered as eye drops, with a concentration of 1 U/mL, four times per day. Notably, no adverse effects resulting from the application of insulin eye drops were observed. The demographic characteristics of the patients treated with insulin eye drops are presented in Table 1. The patients were categorized into three groups based on the initial epithelial defect size: small (≤ 5.50 mm^2^), medium (5.51–16.00 mm^2^), and large (≥ 16.01 mm^2^). A total of 16 patients, accounting for 69.6% of the sample, achieved complete wound healing within a maximum follow-up period of 50 days. Conversely, 7 patients, representing 30.4% of the sample, did not respond to treatment. In the group of patients with small wounds, topical insulin resulted in a reduction in wound size for 6 out of 8 patients, corresponding to a success rate of 75% within a maximum treatment duration of 20 days. Among patients with moderate wound size, 5 out of 8 individuals, or 61% of the group, experienced a resolution of their PED symptoms within 20–30 days. Additionally, 5 out of 7 patients with larger epithelial defects achieved recovery within a maximum period of approximately 50 days.

**Table 1:** The demographic data of patients and Cox regression model analysis of the risk factors for PED healing.

		Final status		HR*	95% CI		P
		Not Healed	Healed		Lower	Upper	
n (%)		7 (30.4%)	16 (69.6%)	–			
Age (Binned)	< = 40	1 (25.0%)	3 (75.0%)	1.00			0.114
	41–60	3 (37.5%)	5 (62.5%)	0.36	0.07	1.84	0.221
	61+	3 (27.3%)	8 (72.7%)	1.83	0.44	7.56	0.406
Gender	F	3 (30.0%)	7 (70.0%)	1.00			
	M	4 (30.8%)	9 (69.2%)	0.46	0.14	1.50	0.199
Smoke	No	5 (31.3%)	11 (68.8%)	1.00			
	Yes	2 (28.6%)	5 (71.4%)	12.27	1.47	102.31	0.021
Diabetes	No	5 (31.3%)	11 (68.8%)	1.00			
	Yes	2 (28.6%)	5 (71.4%)	0.60	0.20	1.80	0.361
Htn	No	6 (28.6%)	15 (71.4%)	1.00			
	Yes	1 (50.0%)	1 (50.0%)	0.56	0.07	4.58	0.590
Size0 (Binned)	< = 5.50	2 (25.0%)	6 (75.0%)	1.00			0.129
	5.51–16.00	3 (37.5%)	5 (62.5%)	0.46	0.12	1.71	0.244
	16.01+	2 (28.6%)	5 (71.4%)	0.21	0.05	0.96	0.044

*Hazard Ratio, based on Cox regression analysis.

**Figure 1:**
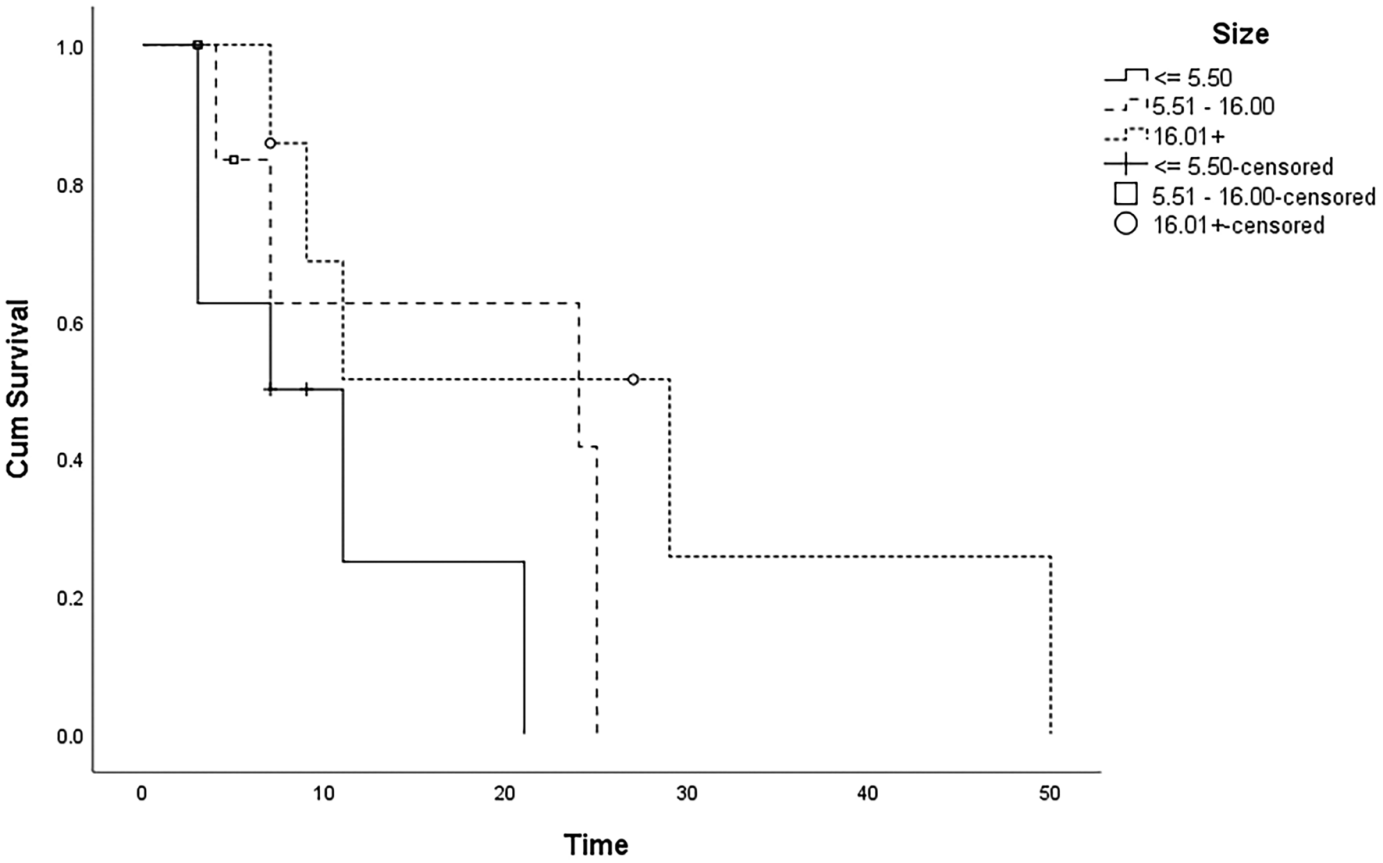
Kaplan–Meier survival plot for PED healing success by days and wound size.

We couldn’t find a specific cause identified for the development of epithelial defects in all patients. Among the cases studied, 5 patients had herpetic keratitis, 2 had a history of anesthetic topical drug abuse, and 2 had facial nerve paralysis. However, due to the limited sample size in each subgroup, the results of the analysis were not statistically significant. For the remaining cases, no specific cause was identified. Nonetheless, we conducted a thorough examination of general risk factors for wound healing, such as diabetes, in all patients. The risk factors for achieving PED healing were analyzed via Kaplan–Meier survival curves and the Cox regression model. Cox regression model analysis identified the initial epithelial defect size as the most significant independent predictor of PED healing in this study (HR for the risk of wound healing: 0.21, 95% CI: 0.96–1.71, P = 0.04). Active smoking was also associated with higher rates of incomplete wound healing in the 50-day Cox regression analysis (HR:12.27, 95% CI: 1.47–1023.3, P = 0.02).Some baseline characteristics however, including age, gender, diabetes and hypertension, did not exhibit any significant correlation with the duration of healing in the univariable analysis (Table 1). Kaplan–Meier survival plot illustrating 50-day PED healing success based on the healing duration and wound size, showed quicker wound healing for patients with PEDs ≤ 5.50 mm^2^ who were treated with topical insulin (Fig. 1).

## Discussion

PED is an umbrella term for any non-healing corneal epithelial defect that fails to show rapid re-epithelialization despite 10–14 days of standard treatments. If left untreated, PEDs can cause significant pain^17^ and discomfort and a wide range of sight-threatening conditions, such as disorganized extracellular matrix, deep corneal ulcer, stromal melting, and scarring. Since prompt epithelial homeostasis restoration upon alteration by disease or trauma is vital for the successful management of epithelial defects and maintaining the cornea’s integrity and mechanical barrier function, effective treatment of PEDs refractory to conventional therapy remains a challenging task for ophthalmologists^18^. In view of therapeutic strategies, despite the relative success of the current stepwise treatment plan for managing PEDs, specialists acknowledge the need for more affordable, available, and tolerable treatment options with long-lasting efficacy and the fewest side effects possible^4,19^.

Insulin is a naturally occurring bioactive compound closely related to insulin growth factor (IGF) as well as a well-established systemic drug implicated in wound healing by regulating cell growth, proliferation, and metabolism. Primarily, rodent animal models have been used for the preclinical testing of the safety and efficacy of ocular insulin delivery in treating PEDs. In a diabetic rat model of persistent corneal epithelial wound healing, Zagon et al.^13^ revealed that topical insulin supplementation remarkably recovers corneal sensitivity and facilitates re-epithelization of the injured cornea, implying early reversibility of diabetic sensory neuropathy. A more recent experiment in Streptozotocin-induced diabetic rats both in vitro and in vivo indicated that enhanced wound healing promotion by insulin is associated with phosphorylation of insulin receptor substrate 1 (IRS1) in epithelial cells, and subsequent upregulation of Wnt/β-catenin signaling pathway in corneal epithelial tissue and mouse corneal epithelial stem/progenitor cells. Numerous studies have highlighted a complex interaction between the insulin and Wnt signaling pathways, reflecting the physiological basis for the direct action of insulin in wound-healing responses and sensation recovery in the injured corneal epithelium^20,21^.

However, the existing understanding of the effectiveness of topical insulin in humans is primarily derived from retrospective clinical trials conducted on diabetic patients with corneal epithelial defects that did not respond to surgical and pharmacological interventions. In a randomized, placebo-controlled, dose–response study of the safety and efficacy of topical insulin in 32 diabetic patients with a postoperative corneal epithelial defect after vitreoretinal surgery, insulin eye drop of 0.5 U/drop conferred the most substantial efficacy by achieving a 100% healing rate within 72 h compared with placebo and higher concentrations. Although no adverse effect or allergic reaction to topical insulin was reported, the lack of beneficial effects of increasing insulin concentration was associated with reduced migration of epithelial cells during the healing process due to higher toxicity^22^. More relevant to our study are the results obtained by Wang et al.^23^, who conducted a study on six patients, both diabetic and non-diabetic, with refractory neurotrophic corneal ulcers. The patients were treated with topical insulin drops, and the study reported that all six patients achieved complete corneal re-epithelization within a range of 7–25 days. Pascua et al.^24^ also reported 5 cases of refractory corneal epithelial defects that were treated successfully with topical insulin drops.

Here, we prospectively examined the efficacy of PED treatment with insulin eye drops in daily clinical practice. Topical insulin 1 U/mL treatment increased and accelerated corneal re-epithelization, even in patients with significant comorbid conditions. During the follow-up period (50 days maximum), 16 (69.6%) of 23 PED patients revealed a consistent and clinically meaningful improvement in wound size. The results indicated that 75% of patients in the small wound group and 61% of patients in the medium wound group showed evidence of improvement within 20 days of receiving topical insulin 1 IU/mL four times a day. Significantly, the eyes of 71% of patients in the large wound group had effectively re-epithelized defect by the end of at most 50-day follow-up period. This clinical trial yields promising outcomes regarding the utilization of topical insulin for the successful treatment of persistent epithelial defects (PEDs) in patients, regardless of their diabetic status, who have not responded to conventional therapies. Interestingly 68% of non-diabetic patients and 71% of diabetic patients showed a significant reduction in wound size in response to insulin eye drops. We anticipated that risk factors such as smoking or diabetes would have a significant impact on the healing process, as each had two out of seven of non-healed patients. However, these numbers did not yield significant results in the statistical analysis, possibly due to the small sample size in each group. In addition, age, gender, and hypertension had no significant effect on healing response. This however, could be due to the limitations presented in our study, including small sample size. In conclusion topical insulin represents a safe and effective therapeutic option for PED treatment in patients with or without diabetes.

## Data Availability

The data that support the findings of this study are available on request from the corresponding author.
